# Newly Identified Wild Rice Accessions Conferring High Salt Tolerance Might Use a Tissue Tolerance Mechanism in Leaf

**DOI:** 10.3389/fpls.2018.00417

**Published:** 2018-04-23

**Authors:** Manas R. Prusty, Sung-Ryul Kim, Ricky Vinarao, Frederickson Entila, James Egdane, Maria G. Q. Diaz, Kshirod K. Jena

**Affiliations:** ^1^Strategic Innovation Platform, International Rice Research Institute, Manila, Philippines; ^2^Institute of Biological Sciences, University of the Philippines Los Baños, Los Baños, Philippines

**Keywords:** wild rice, *Oryza sativa*, salt tolerance, Na^+^ exclusion, tissue tolerance

## Abstract

Cultivated rice (*Oryza sativa* L.) is very sensitive to salt stress. So far a few rice landraces have been identified as a source of salt tolerance and utilized in rice improvement. These tolerant lines primarily use Na^+^ exclusion mechanism in root which removes Na^+^ from the xylem stream by membrane Na^+^ and K^+^ transporters, and resulted in low Na^+^ accumulation in shoot. Identification of a new donor source conferring high salt tolerance is imperative. Wild relatives of rice having wide genetic diversity are regarded as a potential source for crop improvement. However, they have been less exploited against salt stress. Here, we simultaneously evaluated all 22 wild *Oryza* species along with the cultivated tolerant lines including Pokkali, Nona Bokra, and FL478, and sensitive check varieties under high salinity (240 mM NaCl). Based on the visual salt injury score, three species (*O*. *alta, O*. *latifolia*, and *O*. *coarctata*) and four species (*O*. *rhizomatis, O*. *eichingeri, O*. *minuta*, and *O*. *grandiglumis*) showed higher and similar level of tolerance compared to the tolerant checks, respectively. All three CCDD genome species exhibited salt tolerance, suggesting that the CCDD genome might possess the common genetic factors for salt tolerance. Physiological and biochemical experiments were conducted using the newly isolated tolerant species together with checks under 180 mM NaCl. Interestingly, all wild species showed high Na^+^ concentration in shoot and low concentration in root unlike the tolerant checks. In addition, the wild-tolerant accessions showed a tendency of a high tissue tolerance in leaf, low malondialdehyde level in shoot, and high retention of chlorophyll in the young leaves. These results suggest that the wild species employ tissue tolerance mechanism to manage salt stress. Gene expression analyses of the key salt tolerance-related genes suggested that high Na^+^ in leaf of wild species might be affected by *OsHKT1;4*-mediated Na^+^ exclusion in leaf and the following Na^+^ sequestration in leaf might be occurring independent of tonoplast-localized OsNHX1. The newly isolated wild rice accessions will be valuable materials for both rice improvement to salinity stress and the study of salt tolerance mechanism in plants.

## Introduction

The increasing trend of Na^+^ in the agricultural land is a global threat and a major concern for food security (Yamaguchi and Blumwald, 2005; Shahbaz and Ashraf, 2013). Worldwide, approximately 830 million hectares (ha) of the land is affected from soil salinization and bears an annual loss of US$ 12–27.3 billion due to reductions in crop productivity (Qadir et al., 2014). Further the continued practice of poor irrigation system with improper drainage in the agricultural land and due to changing climate events, 50% of all arable land is expected to be impacted by salinity by 2050 (Wang et al., 2003; Rengasamy, 2010; Assaha et al., 2017). Salinity is measured in terms of electrical conductivity and is defined as saline when the value exceeds a threshold of 4 dSm^-1^ (Munns and Tester, 2008). The effect of salinity stress is brought to the plant in two phases over the time scale, the osmotic stress which is immediately felt by the plants soon after exposure to salt solution and the later ionic stress (Al-Tamimi et al., 2016). In the osmotic stress, plant experience a limited supply of water and solute as a result of NaCl-induced reduction in solute potential in soil. The ionic stress phase is initiated once the Na^+^ from the soil enters the plant. Na is a nonessential element for plant (except in some C_4_ plants) (Nieves-Cordones et al., 2010; Kronzucker et al., 2013) and its elevated level within plant tissues interfere with K^+^ function. K^+^ participates in a series of enzymatic reaction linked to vital metabolic pathways and the high external Na^+^ competes for K^+^ and inhibits its activity and disturbs the cellular homeostasis. Hence, maintenance of a low Na^+^/K^+^ in a plant cell is considered to be a key salt-tolerant trait (Shabala and Pottosin, 2014; Munns et al., 2016). To keep the cytosolic Na^+^ at low level and to maintain osmotic balance, plants employ several mechanisms controlled by the regulation of different physiological, biochemical, and molecular processes at various level of plant structural organization (Flowers, 2004).

Rice (*Oryza sativa*), the dietary staple food for more than half of the world population is a salt sensitive crop (Munns and Tester, 2008). Particularly, the seedling and reproductive stages of rice growth are critically affected by salinity. Rice yield starts to decline beyond a threshold EC of 3 dSm^-1^ with 12% reduction in yield per unit rise in EC (Chinnusamy et al., 2005; Reddy et al., 2014). Therefore, most of the modern high yielding varieties experience up to 50% yield reduction under salt stress of 6 dSm^-1^ and they become totally unproductive beyond 12 dSm^-1^ (Linh et al., 2012). Hence, there is an urgent need to develop salt-tolerant rice cultivar either by breeding or biotechnology approach to sustain rice production. Rice plants mainly employ three mechanisms, ion exclusion, osmotic tolerance, and tissue tolerance to adapt in salt stress (Munns and Tester, 2008; Roy et al., 2014). These mechanisms are brought in to play during the various stages of Na^+^ uptake from soil and its translocation to shoot. The solutes and water from the soil can reach xylem via a symplastic or apoplastic route. In species-like rice, a significant amount (50% of the total Na^+^ uptake) of Na^+^ transport is mediated through apoplastic route, i.e., the movement occurs mainly through intracellular spaces which are also called as “bypass flow” (Horie et al., 2012). Plant salt tolerance at this level can be contributed by casparian strips and suberin layers present in the root endodermal and exodemal layers which act as barriers to this bypass flow (Chen et al., 2011). In the root tip and in the initiation site of lateral roots, these structures are partially effective and hence are the suitable sites for Na^+^ entry into the stele. On the other hand in the symplastic route, Na^+^ is transported radially into the stellar region, loaded in to xylem and finally reaches the shoot by the transpiration stream. In this case the plant salt tolerance can be achieved by the regulation of the transporters localized in the cells of cortex, pericycle, and xylem parenchyama bordering xylem. *SOS1* is a plasma membrane anti-porter present in the root epidermis provides the resistance to Na^+^ uptake by excluding Na^+^ to external environment (Shi et al., 2000). However, the function of *SOS1* depends on the severity of the salt stress. The *SOS1* gene in the Arabidosis (*AtSOS1*) may function both in Na^+^ loading (low or moderate level of salinity) and unloading (high salinity) (Olías et al., 2009; Yadav et al., 2012). The over-expression of rice transporter *OsSOS1* in Arabidopsis has been shown to increase salt tolerance (Martínez-Atienza et al., 2007). It is also known that lower expression of *OsSOS1* in rice old leaves may decrease frequency of retrieving Na^+^ from old leaf cells (Wang et al., 2012). Rice HKT family transporter, *OsHK2;1* present in the plasma membrane of epidermis mediates Na^+^ transport to the root; however, in the cortical region it prevents the radial flow of Na^+^ and restricts its movement to the xylem (Horie et al., 2007). Similarly, *OsHKT1;5* localized in the xylem parenchyma retrieves Na^+^ from xylem sap to xylem parenchyma (Ren et al., 2005). Transporters like *OsHKT2;1, OsHKT1;5*, and *OsSOS1* are the control points in the root soil boundary and their regulation decides the fate of Na^+^ entry in to the xylem (Zhang et al., 2017). Once Na^+^ is loaded to xylem it is transported via transpiration pull to the shoot. In monocot species like rice *OsHKT1;4* transporter, located in the leaf sheath region can further retrieve the Na^+^ from the xylem to xylem parenchyma lowering their delivery to the leaf blade (Suzuki et al., 2016). Most of the metabolic process of the plant is carried in the leaf blade and hence it is needed to be kept away from reaching a toxic concentration of Na^+^. Re-circulation of Na^+^ from the leaf/shoot to the root via phloem is a possible mechanism for salt tolerance and which occurs in plants like Arabidopsis and reeds but in rice it is not a well-established mechanism (Berthomieu et al., 2003; Horie et al., 2005). However, *OsHKT2;1* localized to the shoot vascular bundle is believed to recirculate Na^+^ to the root via phloem (Golldack et al., 2002; Laurie et al., 2002). There are certain salt-tolerant glycophytes and halophytes that are able to grow in high salt concentration (>200 mM NaCL) and their tissues can also tolerate equivalent concentration of Na^+^ (Flowers and Colmer, 2008; Katschnig et al., 2015). The Na^+^ in their tissue acts like an osmoticum to adjust the osmotic pressure and they are not completely dependent on the synthesis of organic solute which is energetically expensive. The capacity of the tissue to function while containing a high internal concentration of Na^+^ is known as tissue tolerance. Intracellular compartmentalization of Na^+^ in to the vacuole by tonoplast Na^+^/H^+^ exchanger, *NHX* is a key mechanism of tissue tolerance for which the Na^+^ concentration remains relatively low in the cytoplasm. However, other factors like synthesis of compatible solutes, synthesis of enzymes for catalyzing detoxification of ROS, and maintenance of cell volume and turgor also contribute to tissue tolerance (Munns et al., 2016).

Salt tolerance in rice primarily depends on Na^+^ exclusion principle. Most of the salt-tolerant accessions of cultivated rice maintain a low Na^+^ concentration in the actively growing plant parts. *OsHKT1;*5 in rice is a major determinant for salt tolerance. The activity of *OsHKT1;5* is more robust in salt-tolerant rice cultivars and was originally detected from a salt-tolerant land race Nona Bokra (Bonilla et al., 2002; Ren et al., 2005). It is also believed to be the causal gene in *Saltol* QTL region which accounts important aspect of seedling salt tolerance (Bonilla et al., 2002; Lin et al., 2004). Cultivated rice has a narrow genetic diversity for salt tolerance with a less number of donor source, limited to traditional rice land races only. Due to this limitation, the rice breeding program has no alternative choice rather using these donors repeatedly for improving salt tolerance in rice varieties. (Thomson et al., 2010; Platten et al., 2013; Reddy et al., 2014). Identification of diverse germplasm with high salt tolerance is an imperative and important strategy to adapt to a high salinity projected from the current climate change. The wild relatives of rice offer an untapped genetic resource of novel genes with diverse sets of adaptive mechanisms for development of climate resilient rice cultivar (Brar and Khush, 1997). These germplasm has to be exploited in depth to find suitable tolerance source and to further use it in rice breeding. There are 22 wild species in the rice genus along with two cultivated rice (Marathi et al., 2014). Around 4,370 accessions of wild species are available at the gene bank of IRRI and are grouped into 11 different genome types (AA, BB, CC, BBCC, CCDD, EE, FF, GG, HHJJ, HHKK, and KKLL) (Jena, 2010; Sanchez et al., 2013). So far salt tolerance is of concern, and a few wild germplasm are known to be salt tolerant with unknown salt tolerance mechanism. This study attempts to evaluate the wild rice accessions from all genomes of wild rice species to isolate promising salt-tolerant sources and to understand the salt tolerance mechanism of different genomes.

## Materials and Methods

### Plant Materials and Preparation of Seedlings

A total of 22 different wild *Oryza* species (one accession per species) with salt-tolerant checks (Pokkali, Nona Bokra, and FL478) and salt-sensitive checks (IR29 and IR75862-206-2-8-3) were used in this study. Seeds of these materials were obtained from the International Rice Genebank of IRRI. Seeds were kept in a convection oven at 50°C for 5 days for dormancy breaking and were dehusked. The seeds were sterilized by treatment of 70% ethanol for 1 min and 1.5% sodium hypochlorite solution for 30 min. After which, the seeds were washed with sterilized water for 5 times. Seeds were transferred into culture tubes with one quarter-strength of Murashige and Skoog media and kept in the dark room for 48 h to obtain uniform germination. Once the seeds started sprouting, the culture tubes were kept under light condition for 5 days and the plantlets were transferred to seedling floats in a tray containing Yoshida nutrient solution (Yoshida et al., 1976). For curing of any plant damage during seedling transfer, the trays were kept for 3 days in Yoshida solution at the phytotron plant growth facility of IRRI. Seeds of *O. coarctata, O*. *schlechteri*, and *O*. *meyeriana* were hard to germinate, hence the seedlings were established in nutrient solution by cutting internodes.

### Plant Growth and Salt Stress Treatment

For salinization, initially 60 mM NaCl (EC6) in Yoshida solution was applied to the seedling plants after curing and an increment of 60 mM NaCl was carried at an interval of 2 days until 180 mM NaCl for physiological and biochemical analyses and 240 mM NaCl for the determination of days of seedling survival (DSS). Nutrient solutions containing salt (NaCl) were replaced every 5 days and the solution was maintained with a pH of 5.0 daily. As a control condition, an identical set of seedlings were continuously kept in a normal Yoshida solution.

### Determination of Days of Seedling Survival

The DSS was determined by counting the number of days the seedling survived under 240 mM NaCl condition. When the plants were completely wilted, it was regarded as dead. The mean values of DSS were obtained from the two independent experiments (eight plants per entry in each experiment).

### Visual Salt Injury (VSI)

The VSI score for different entries were determined after 16 days of salinization in 180 mM NaCl. VSI scores were given following the standard evaluation system (SES) of IRRI (Gregorio et al., 1997). Briefly, the scores of 1, 3, 5, 7, and 9 were assigned for highly tolerant, tolerant, moderately tolerant, sensitive, and highly sensitive, respectively to salt stress. Mean values were calculated from the two replications. In each experiment, eight plants per entry were scored.

### Plant Vigor

To test the vigor (VI) of growth, all entries were grown in normal Yoshida solution for 20 days. Then, shoots of each plant were harvested for the measurement of fresh biomass and shoot length. Mean values were obtained from the five plants per entry. The difference obtained between the maximum and minimum mean value of the entries were divided by 5 and all entries were assigned into five groups with scores from 1, 3, 5, 7 to 9 (from high to low VI score).

### Plant Growth Reduction by Salt Stress

Shoot length and biomass were compared between the control and salt stress condition (180 mM NaCl) and plant growth reduction by salt stress was represented in percentage. The mean values were obtained from five plants per entry in the normal and salt stress conditions, respectively.

### Quantification of Malondialdehyde

To assess the damage incurred from the stress, malondialdehyde (MDA) levels were measured from leaf tissues following Hodges et al. (1999) with minor modifications. Briefly, leaf tissues were harvested from the plants after 10 days of salt treatment (180 mM NaCl) and homogenized in liquid N_2_. 0.5 g of the homogenized leaf was mixed with 0.1% (w/v) trichloroacetic acid solution and the homogenate was centrifuged at 4,000 × *g* for 15 min at 4°C. Aliquots of 1 mL of the supernatant were placed in two tubes, one with thiobarbituric acid (TBA) reagent and another without TBA reagent. The mixture was incubated at 90°C for 25 min and the reaction was stopped by placing the tubes in an ice bath. Absorbance of the supernatant was obtained at 440, 532, and 600 nm and MDA estimates were computed and expressed in fresh weight basis. The MDA values were obtained from the average of three plants per entry.

### Chlorophyll Quantification in Young Emerging Leaf

To quantify chlorotic symptoms due to salinity stress, chlorophyll content was determined following Lichtenthaler and Buschmann (2001) with slight modifications. Young emerging leaves (L6) were collected 10 days after salinization with 180 mM NaCl and were temporarily stored at -80°C until assay. Ten milligrams of the homogenized leaf samples were placed into 10 ml of 95% ethanol and were incubated at 80°C for 10 min, cooled at room temperature thereafter, and then reconstituted to the original volume by adding 95% ethanol. Absorbance values were obtained at 470, 649, and 664 nm using spectrophotometer. The chlorophyll concentrations were determined by following the equation of Lichtenthaler and Buschmann (2001). Total chlorophyll content was measured from five plants per accession in the control and 180 mM NaCl conditions.

### Measurement of Ion Content in Leaf and Root

Shoot and root were harvested at 16 days after salt treatment (180 mM) and were dried in an oven (65°C) for 48 h. Approximately 10 mg was weighed, placed in 50 ml conical tubes and digested by 10 ml of 0.1 N acetic acid (Sigma-Aldrich, United States) at 90°C for 2 h. The extracts were cooled at room temperature, left overnight, and then filtered using Whatman filter paper. Finally, Na^+^ and K^+^ ions were measured using a PerkinElmer AAnalyst 200 atomic absorption spectrophotometer (PerkinElmer, United States), operating in emission mode. Mean values of Na^+^ and K^+^ contents in both root and leaf were obtained from five plants per entry.

### Tissue Tolerance Assay

Tissue tolerance assay is an indirect way of estimating the process of Na^+^ sequestration (Yeo et al., 1990). It was conducted for the specific wild salt-tolerant species and the check lines. The same sets of genotypes were treated to a series of salt concentrations (0, 60, 120, 180, and 240 mM NaCl). Initially, 60 mM NaCl was given to all plants in the setup and an increment of 60 mM NaCl was applied at an interval of 2 days to reach higher salt concentrations. The phenotype of each material under different salt concentrations was monitored through the observation of VSI scores based on the IRRI SES (Gregorio et al., 1997). Once a genotype reached an SES score of 7 at the highest salinity level (240 mM), the youngest fully expanded leaf (6th Leaf, L6) was harvested from each salt concentration. The experiment was continued until all the genotypes were harvested and the harvested samples were analyzed for Na^+^ content and chlorophyll content. The tissue tolerance score was estimated from the LC_50_ score which represents the Na^+^ content at which 50% reduction of the chlorophyll content occurs. Mean value obtained from two replications (four plants per entry in each replication) were used for determining the LC_50_ scores.

### Measurement of Na^+^ Content of Leaf Surface

Two to three leaves were detached from a similar position in each plant after 10 days at 180 mM NaCl and were carefully put in a tube containing 10 ml of double distilled water. The tubes were shaken vigorously for 2 min to wash the leaf surface properly. After washing, the solution was used for Na^+^ quantification and the leaf tissues were scanned to measure the total leaf surface area. Na^+^ content per unit leaf area was calculated from five plants per accession.

### Leaf Anatomy

Leaf tissues were detached from all wild tolerant genotypes and were fixed in glacial acetic acid–ethanol (1:3) solution for 24–48 h. Transverse sections of the leaf samples were made using a razor blade and stained with 1% toluidine blue solution. The specimen was observed under a light microscope (Olympus BX53).

### Gene Expression Analysis of the Known Salt Tolerance Genes

A floating tray containing 20-day-old seedling plants in Yoshida solution was transferred to the salt-added Yoshida solution with the following concentrations: 80 mM NaCl for 8 h, 160 mM NaCl for 16 h, and 240 mM NaCl for 24 h. Leaf and root tissues were then collected from the normal and the salt-treated conditions, respectively. Each sample tube contained the tissues derived from 2 to 4 seedling plants and three tubes per experiment were prepared. Total RNA was extracted and cDNA was synthesized using an ImProm-II Reverse Transcription system (Promega, United States). The expression of salt-tolerant genes including *OsNHXl, OsHKTl;4, OsHKTl;5*, and *OsSOSl* were performed using SYBR select master mix and ABI7500 machine (Applied Biosystems, United States). The primer sequences for the target genes are listed in Supplementary Table S1 (Additional File 1). The *OsActl* gene was used as an internal control and the relative expression level was calculated based on the ΔΔ*Ct* method. Each data point represents the mean value of three biological replications.

## Results

### Wild Rice Species Possesses Higher Tolerance to Salinity

**FIGURE 1:**
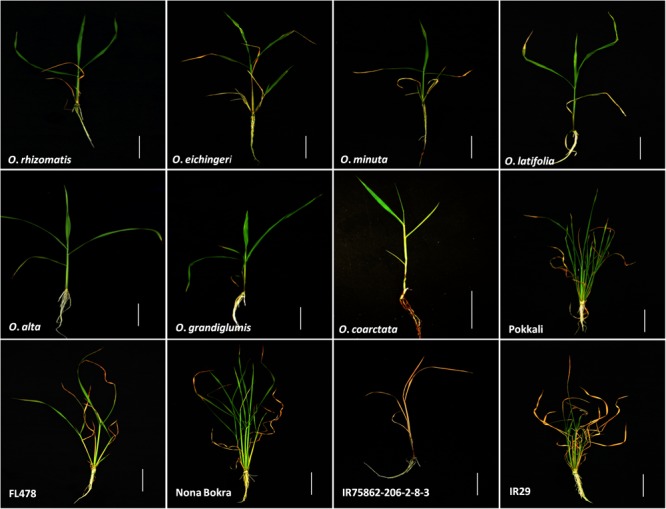
Seedling phenotype of tolerant wild species with cultivated checks after 10 days of 180 mM NaCl treatment. Scale bar = 5 cm.

**FIGURE 2:**
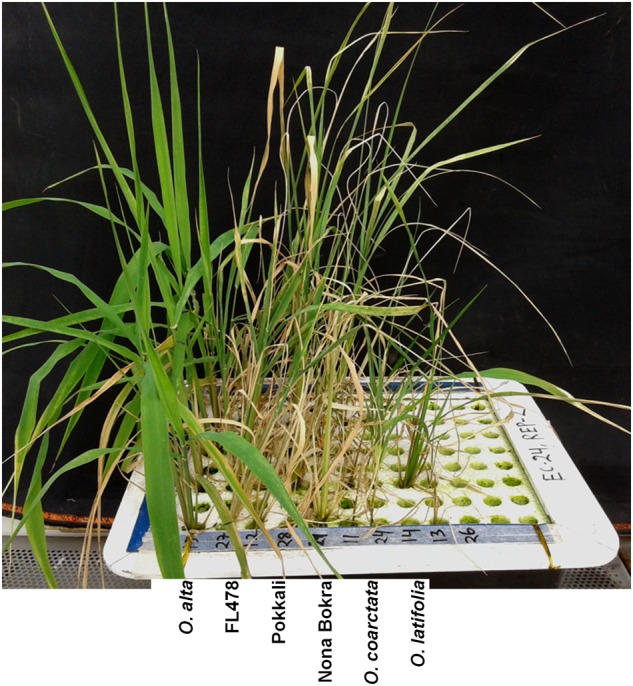
Seedlings phenotype of the three highly tolerant wild species with the tolerant check lines after 10 days of 240 mM NaCl treatment. Scale bar = 5 cm.

To isolate new salt-tolerant germplasm, representative from 24 *Oryza* species together with tolerant and sensitive checks were exposed to salt stress. The wild rice accessions showed various degrees of tolerance to salt stress at both 180 and 240 mM of NaCl (**Table 1** and **Figure 1**) compared to control condition (Additional File 2: Supplementary Figure S1). Based on the plant phenotype under salt stress as rated by the VSI score, five wild *Oryza* species (*O*. *nivara, O*. *brachyantha, O*. *granulata, O*. *longiglumis, O*. *rufipogon*, and *O*. *schlechteri*) were classified as highly salt sensitive like the susceptible check cultivars (IR29 and IR75862-206-2-8-3) and these species survived only for <5 days at 240 mM NaCl (DSS score 2.8–4.6). Seven wild species accessions (*O*. *meridionalis, O*. *glumaepatula, O*. *australiensis, O*. *ridleyi*, and *O*. *meyeriana*) were classified as the sensitive group and they survived for 5–7 days. The five wild species accessions (*O*. *punctata, O*. *barthii, O*. *officinalis, O*. *longistaminata*, and *O*. *glaberrima*) and one rice cultivar (IR64) showed moderate level of salt tolerance (VSI score 5.0–6.6) and their DSS was in the range of 5.6–8.6 days. Four wild species accessions (*O*. *rhizomatis, O*. *eichingeri, O*. *minuta*, and *O*. *grandiglumis*) along with the three tolerant check cultivars (FL478, Pokkali, and Nona Bokra) belonged to the tolerant group and they survived upto 16 days at 240 mM NaCl (DSS score 10.3–15.8). The wild species, *O. latifolia, O. alta*, and *O*. *coarctata* showed high salt tolerance. *O*. *coarctata* was the most tolerant wild species as it survived during the seedling stage without any detrimental effect and grew up to reproductive stage. The species, *O*. *alta* which was found to be the second most highly tolerant species had a DSS of 33 days in 240 mM NaCl. The DSS of *O*. *latifolia, O*. *alta*, and *O*. *coarctata* was significantly higher (*p* < 0.001) than the tolerant check cultivars FL478, Pokkali, and Nona Bokra. Visual difference in tolerance between the wild-tolerant species (*O*. *alta, O*. *latifolia*, and *O*. *coarctata*) and the conventional tolerant checks is presented in **Figure 2**. On the basis of DSS, salt tolerance of all tolerant genotypes was ranked in the following order: *O*. *coarctata, O*. *alta, O*. *latifolia, O*. *grandiglumis, O*. *minuta*, Nona Bokra, Pokkali, FL478, *O*. *eichingeri*, and *O. rhizomatis*.

**Table 1:** Salt stress phenotypes as determined by VSI score and days of seeding survival (DSS).

Species	Accessions (IRGC No.)/variety	Genome	VSI score (*M* ±*SE*)	VSI group^+^	DSS	VS score^++^
*O*. *brachyantha*	Acc.101232	FF	9.0^a^	HS	2.8 ± 0.09^q^	9^a^
*O*. *granulata*	Acc.102118	GG	9.0^a^	HS	2.8 ± 0.18^q^	9^a^
*O*. *longiglumis*	Acc.105148	HHJJ	9.0^a^	HS	2.8 ± 0.09^q^	8^b^
*O*. *schlecteri*	Acc.82047	HHKK	9.0^a^	HS	3.3 ± 0.12^pq^	9^a^
*O. sativa*	Acc.IR75862-206-2-8-3	AA	9.0^a^	HS	3.0 ± 0.17^pq^	7.2^c^
*O. sativa*	IR29	AA	8.1 ± 0.24^b^	HS	4.6 ± 0.12^l^	7^cd^
*O*. *nivara*	Acc.80455	AA	9.0^a^	HS	4 ± 0.15^mn^	5.4g
*O*. *rufipogon*	Acc.80671	AA	8.2 ± 0.24^b^	HS	3.5 ± 0.19^nop^	6.2^ef^
*O*. *ridleyi*	Acc.100821	HHJJ	7.8 ± 0.24^bc^	S	4.9 ± 0.27^i^	9^a^
*O*. *meridionalis*	Acc.105301	AA	7.8 ± 0.24^bc^	S	4.6 ± 0.19^l^	7^cd^
*O*. *glumaepatula*	Acc.105692	AA	7.8 ± 0.24^bc^	S	4.4 ± 0.12^lm^	7^cd^
*O*. *australiensis*	Acc.100882	EE	7.8 ± 0.24^bc^	S	3.7 ± 0.10^no^	8^b^
*O*. *meyeriana*	Acc.89241	GG	7.3 ± 0.19^c^	S	5.6 ± 0.12^K^	9^a^
*O*. *longistaminata*	Acc.110404	AA	6.2 ± 0.24^de^	MT	6.6 ± 0.27^j^	3^i^
*O*. *punctata*	Acc.105690	BB	5.0^gh^	MT	8.6 ± 0.12^h^	6^f^
*O. sativa*	IR64	AA	5.8 ± 0.24^ef^	MT	7.5 ± .0.21^i^	5.2^g^
*O*. *barthii*	Acc.100936	AA	6.6 ± 0.19^d^	MT	5.6 ± 0.19^k^	7.2^c^
*O*. *glaberrima*	Acc.96717	AA	6.6 ± 0.19^d^	MT	5.6 ± 0.19^k^	6^f^
*O*. *officinalis*	Acc.100896	CC	5.3 ± 0.19^fg^	MT	7.4 ± 0.24^i^	8^b^
*O*. *rhizomatis*	Acc.105432	CC	4.6 ± 0.19^hi^	T	10.3 ± 0.24^g^	8^b^
*O*. *eichingeri*	Acc.101424	CC	4.3 ± 0.23^i^	T	11.5 ± 0.26^f^	6.8^cd^
*O*. *grandiglumis*	Acc.101405	CCDD	3.7 ± 0.24^jk^	T	15.8 ± 0.18^c^	6.6^de^
*O*. *minuta*	Acc.101141	BBCC	4.1 ± 0.24^ij^	T	15.8 ± 0.22^c^	9^a^
*O. sativa*	FL478	AA	4.3 ± 0.23^i^	T	11.8 ± 0.28^f^	4.4^h^
*O. sativa*	Pokkali	AA	4.2 ± 0.24^ij^	T	13.1 ± 0.09^e^	1^j^
*O. sativa*	Nona Bokra	AA	4.1 ± 0.24^ij^	T	14.8 ± 0.20^d^	4.2^h^
*O*. *latifolia*	Acc.105133	CCDD	2.8 ± 0.21^k^	HT	26.1 ± 0.24^b^	7.2^c^
*O*. *alta*	Acc.105143	CCDD	1.3 ± 0.19^l^	HT	33 ± 0.21^a^	7^cd^
*O*. *coarctata*	Acc.104502	KKLL	1.0^l^	HT	^∗^	9^a^

*^+^HS, highly sensitive; S, sensitive; MT, moderately tolerant; T, tolerant; HT, highly tolerant. VSI is determined following IRRI SES and was obtained after 10 days of growth at 180 mM NaCl. VSI score of 8.0–9.0 = HS; 7.0–7.9 = S; 5.0–6.9 = MT; 3.0–4.9 = T; and <2.9 = HT. The DSS was obtained at 240 mM NaCl. ^++^VS of different genotypes is determined from their growth rate in nonsaline nutrient solution. Mean value with different letteres statistically different at *p* < (0.05) based on DMRT.*

Plant vigor is positively associated with salt tolerance in rice species. To check the plant vigor in this study, we measured shoot biomass and shoot length of all the test material after 20 days of their growth in normal condition (non-saline nutrient solution). The cultivated checks especially Pokkali and Nona Bokra grew faster than the wild accessions and showed vigorous growth (VS score 1.0 and 4.2, respectively) (**Table 1**). The wild species-tolerant lines grew slower than the cultivated check during the same 20 days of observational period. The vigor score of the wild species-tolerant lines differed significantly (*p* < 0.001) from that of cultivated tolerant lines. The correlation of vigor score with salt tolerance was high (*r*^2^ = 0.90) for cultivated rice lines whereas it was low for wild species (*r*^2^ = 0.026).

Shoot part is mostly affected in salt stress than the root. To observe the effect of salinity on shoot growth, shoot length and shoot biomass were measured from the newly identified tolerant species along with the check lines (**Table 2**). Except for *O*. *coarctata*, a significant reduction in shoot length and shoot biomass was observed in all the wild and cultivated check lines. Among the tolerant lines, Nona Bokra experienced least reduction in shoot length (16.28%) and *O*. *alta* encountered least reduction (30.60%) in shoot biomass. While the highest reductions of both shoot length (34.02%) and biomass (53.24%) occurred in *O*. *rhizomatis.* The susceptible checks (IR75862-206-2-8-3 and IR29) showed dramatic growth inhibition by salt stress (54.89 and 45.73% in shoot length and 85.10 and 66.0% in biomass, respectively). *O*. *coarctata* did not encounter any reduction in growth parameter under salt concentration rather its shoot length and shoot biomass increased as 10.45 and 11.6%, respectively, in saline condition.

**Table 2:** Reduction in the shoot biomass and shoot length of the salt-tolerant wild accessions and check lines at 180 mM NaCl.

Genotypes	Shoot length (cm)			Shoot biomass (g)		
	Control	180 mM NaCl	% Reduction from control	Control	180 mM NaCl	% Reduction from control
*O. rhizomatis*	31.6 ± 0.96	20.34 ± 0.31	34.02^bc^	0.74 ± 0.05	0.34 ± 0.02	53.24^c^
*O. eichingeri*	55.1 ± 0.13	36.55 ± 1.10	34.35^bc^	2.73 ± 0.06	1.32 ± 0.05	52.57^c^
*O. minuta*	21.8 ± 1.15	17.17 ± 0.55	20.32^de^	0.87 ± 0.04	0.66 ± 0.02	26.9^e^
*O. latifolia*	48 ± 1.81	36.22 ± 1.42	24.79^cde^	2.53 ± 0.10	1.52 ± 0.02	47^cd^
*O. alta*	51.2 ± 0.91	39.57 ± 0.95	23.06^cde^	2.22 ± 0.08	1.75 ± 0.05	30.6^e^
*O. grandiglumis*	59.7 ± 1.19	41.17 ± 1.19	30.23^cd^	3.13 ± 0.44	1.75 ± 0.04	49.7^c^
*O. coarctata*	13.4 ± 0.83	14.65 ± 0.40	–10.45^f^	0.28 ± 0.01	0.39 ± 0.08	–11.6^f^
IR75862-206-2-8-3	47 ± 1.52	21.13 ± 0.33	54.89^a^	3.08 ± 0.06	0.39 ± 0.08	85.10^a^
IR29	50.2 ± 1.11	27.67 ± 0.69	45.73^ab^	2.92 ± 0.07	0.96 ± 0.02	66^b^
FL478	64.1 ± 0.98	44.80 ± 0.54	24.49^cde^	8.24 ± 0.48	4.91 ± 0.25	37.2^de^
Pokkali	89.5 ± 0.89	66.50 ± 0.69	19.26^de^	17.42 ± 0.45	10.5 ± 0.28	37.6^de^
Nona Bokra	75.8 ± 1.58	57.17 ± 0.93	16.28^e^	8.38 ± 0.45	5.40 ± 0.26	36.9^de^

*The data represented are mean values (*n = 5*) with standard errors. Different letters in the % reduction coulmn are statistically different as inferred from DMRT at *p* < (0.05).*

### Wild-Tolerant Species Sustained Minimal Cell Injury Under Salinity Stress

**FIGURE 3:**
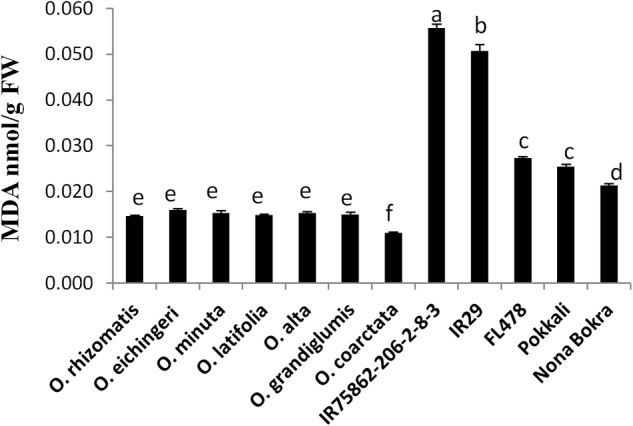
Leaf MDA content of tolerant wild species and the cultivated checks. Leaf tissues were collected after 10 days from a 180 mM NaCl-treated experimental set up and mean value of MDA (*n* = 3) was calculated in nmol per gram FW of the leaf. Vertical bar indicates ± standard error. Different letters in error bars are statistically different as inferred from DMRT at *p* < 0.05.

Oxidative damage is regarded as the manifestation of stress susceptibility and one of its causes is extensive membrane lipid peroxidation. MDA level is widely used as an indicator of the extent of oxidation damage under salt stress. MDA content of shoot was measured from the tolerant wild species and the check lines which were stressed by 180 mM NaCl. The sensitive checks IR29 and IR75862-206-2-8-3 showed high MDA content in the shoot, indicating a higher degree of lipid peroxidation and cell membrane damage while both cultivated and wild-tolerant species contained low level of MDA (**Figure 3**). Among the tolerant lines, MDA content was significantly lower (0.4- to 1.5-fold) in all wild-tolerant lines than tolerant cultivars which indicates the wild-tolerant lines have a better stress amelioration. The lowest MDA level was obtained for *O. coarctata*.

### Tolerant Wild Species Accessions Showed High Chlorophyll Content in the Young Emerging Leaves Under Salt Stress Condition

Retention of chlorophyll level in the leaves under salt stress is used as an index for salt tolerance. Total chlorophyll content was measured from the young emerging leaves in both normal and salt conditions (**Table 3**). Difference of chlorophyll content between normal and salt conditions was very high (39.16–69.57%) in the sensitive and tolerant checks. In contrast, the salt-tolerant wild species showed relatively low chlorophyll reduction with wide variations (4.59–39.80%). Chlorophyll reduction was very less in *O. eichingeri, O*. *minuta*, and *O. coarctata* (4.50–13.10%).

**Table 3:** Reduction in chlorophyll content in the young leaves (L6) of the salt-tolerant wild accessions and check lines at 180 mM NaCl.

Genotypes	Chlorophyll content		% of reduction^∗^
	Control	180 mM NaCl	
*O*. *rhizomatis*	2.46 ± 0.15	1.48 ± 0.21	39.80^de^
*O*. *eichingeri*	2.78 ± 0.18	2.42 ± 0.19	13.10^g^
*O*. *minuta*	2.85 ± 0.18	2.63 ± 0.19	8.10^h^
*O*. *latifolia*	2.86 ± 0.21	2.08 ± 0.31	37.16^e^
*O*. *alta*	1.82 ± 0.13	1.38 ± 0.18	23.22^f^
*O*. *coarctata*	2.09 ± 0.18	2.00 ± 0.18	4.50^h^
*O*. *grandiglumis*	1.82 ± 0.18	1.20 ± 0.18	36.26^e^
IR75862-206-2-8-3	2.95 ± 0.20	0.86 ± 0.18	69.57^a^
IR29	2.95 ± 0.22	1.90 ± 0.18	39.16^de^
FL478	3.19 ± 0.24	1.88 ± 0.19	50.90^b^
Pokkali	2.79 ± 0.16	1.55 ± 0.15	43.65^cd^
Nona Bokra	2.20 ± 0.15	1.18 ± 0.14	45.81^bc^

*^∗^The data represented are mean values (*n = 5*) with standard errors. Different letters in the % reduction coulmn are statistically different as inferred from DMRT at *p* < (0.05).*

### Tolerant Wild Species Lines Showed High Na^+^ Content in Leaves and Low Accumulated Na^+^ Content in Roots

**FIGURE 4:**
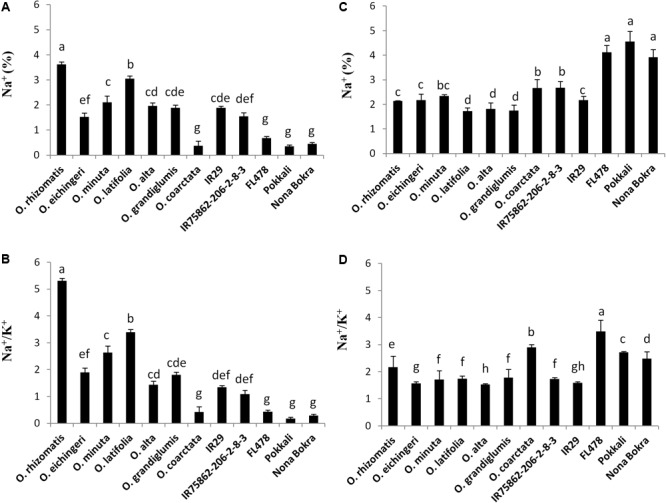
Na^+^ content (%) and Na^+^/K^+^ ratio in shoot **(A,B)** and root **(C,D)**, respectively, in the tolerant wild species and the cultivated checks after 10 days of 180 mM NaCl treatment. Mean values were obtained from five plants. Vertical bars indicates ±SE. Different letters in error bars are statistically different as inferred from DMRT at *p* < 0.05.

Na^+^ exclusion at root xylem epithelial region is considered to be the primary mechanism of salt tolerance in conventional tolerant lines like FL478, Nona Bokra, and Pokkali. Low Na^+^ in the shoot is used as selection criteria to breed salt-tolerant cultivar in wheat, barley, and rice. However, in our study, we observed an opposite trend in shoot Na^+^ and Na^+^/K^+^ pattern between the tolerant wild species (except *O*. *coarctata*) and tolerant cultivars (**Figures 4A,B**). The cultivated tolerant checks and *O*. *coarctata* showed low Na^+^ and low Na^+^/K^+^ in the leaves. Oppositely, Na^+^ and Na^+^/K^+^ ratio in the newly isolated wild-tolerant lines was significantly high even higher than sensitive check lines IR29 and IR75862-206-2-8-3. Similarly to the leaf, a contrasting root ion profile was also observed between the newly identified tolerant wild species and the cultivated tolerant checks. The root Na^+^ content and Na^+^/K^+^ were found to be high in cultivated salt-tolerant checks while it was low in wild-tolerant species and in the sensitive checks (**Figures 4C,D**).

### Gene Conservation and Expression Analyses of the Salt Transporter Genes in the Wild-Tolerant Accessions

Several salt tolerance-involved genes including *OsNHX1, OsHKT1;4, OsHKT1;5*, and *OsSOS1* have been identified and studied in rice. We conducted PCR amplifications of the above genes in tolerant wild species because the species of *Oryza* genus diversified in the ancient time. Three primer sets for *OsNHX1*, two primer sets for *OsHKT1;4*, six primer sets for *OsHKT1*;*5*, and four primer sets for *OsSOS1* were designed (Additional File 1: Supplementary Table S1) and applied for the PCRs with genomic DNA of the wild-tolerant accessions. All four genes tested in this study were amplified in all wild-tolerant species and the checks, except for few sets of primer (Additional File 3: Supplementary Figure S2). This result supports that these four salt-related genes existed in the origin of the *Oryza* genus before diversification of species. In the case of *OsHKT1;4* gene, OsH4F1 primer set showed different PCR band size between wild species and cultivated rice (*O. sativa*) (Additional File 3: Supplementary Figure S2B), suggesting that the *O. sativa* allele might be separated during species differentiation or domestication. In the case of *OsHKT1*;*5* gene, four primer sets locating intron or 5’-untranslated region (UTR) of the gene showed no PCR bands in wild species (Additional File 3: Supplementary Figure S2C), indicating that the sequences of intron or 5’-UTR are highly variable between wild species and *O. sativa*. For *OsSOS1* gene, one primer set (SOS1F2) which has binding site two the exonic regions amplified in case of cultivated rice but did not amplified for wild species (Additional File 3: Supplementary Figure S2D).

**FIGURE 5:**
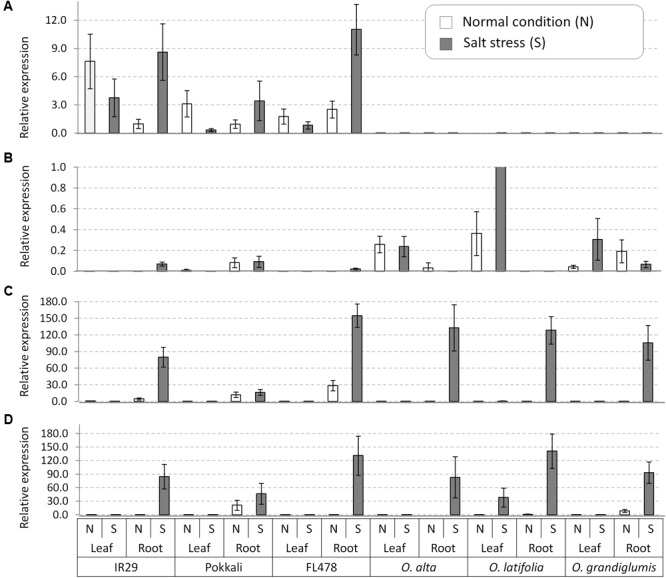
Gene expression analyses of the salt tolerance genes in leaf and root tissues collected from the salt tolerant wild species having the CCDD genomes and the checks varieties. **(A)** *OsNHX1*, **(B)** *OsHKT1;4*, **(C)** *OsHKT1;5*, and **(D)** *OsSOS1*. *OsActil1* was used as an internal control of qRT-PCR. N, normal nutrition solution (white bar); S, salt stress (gray bar). Error bar means SD (*n* = 3).

After confirmation of presence of four genes in the wild species, we conducted gene expression analyses in root and leaf tissues of seedling plants grown under normal and salt-added nutrient solution, respectively. One salt-sensitive check IR29, two conventional tolerant lines Pokkali and FL478, and three CCDD genome species (*O. alta, O. latifolia*, and *O. grandiglumis*) were tested. Transcription of *OsNHX1* increased in root tissue and decreased in leaf tissue in all three cultivated rice under salt stress (**Figure 5A**). In contrast, the *OsNHX1* transcripts were almost non-detectable in all samples of the three CCDD genome species (**Figure 5A**). The expression level of *OsHKT1;4* was relatively high in leaf tissues of all wild species compared to checks in which the gene weakly expressed in root (**Figure 5B**). In the cases of *OsHKT1;5* and *OsSOS1* genes, transcription of both genes was dramatically increased by salt stress in root tissue in all tested materials including salt-sensitive IR29 (**Figures 5C,D**), indicating that these two genes are functioning in root to exclude Na^+^ from xylem in response to salt stress.

### Tolerant Wild Species Lines Showed High Tissue Tolerance in Leaf

**FIGURE 6:**
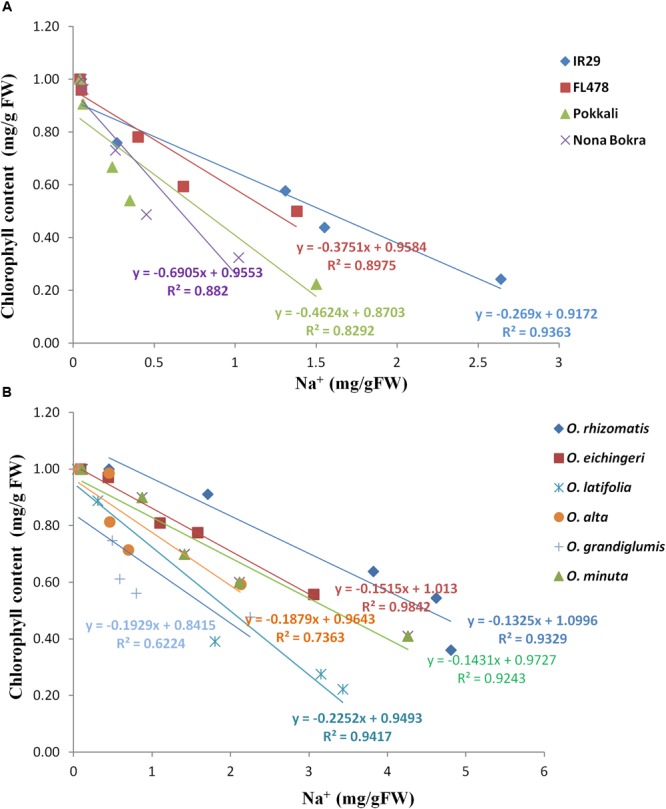
Tissue tolerance score of tolerant wild species and cultivated checks as determined from the LC_50_ value. Check lines **(A)** and wild salt-tolerant lines **(B)**.

Tissue tolerance of a genotype is defined as the capacity of the tissue to function while containing a high concentration of Na^+^. This component of salt tolerance emphasizes the process of Na^+^ sequestration in the vacuole as a result of which metabolic activities in a cell are less affected. Tissue tolerance score (LC_50_) from our experiment for different genotypes was determined from the Na^+^ concentration at which a 50% of the chlorophyll reduction occurs. Low LC_50_ value was obtained for the Na^+^ excluding tolerant cultivars Pokkali (LC_50_ = 0.6 mg/g), Nona Bokra (LC_50_ = 0.9 mg/g), and FL478 (LC_50_ = 1.2 mg/g) (**Figure 6A**), implying that they can undergo rapid chlorophyll loss even at low Na^+^ concentration. The salt-sensitive check IR29 has higher tissue tolerance score (LC_50_ = 1.57 mg/g) than the cultivated salt-tolerant checks. Interestingly, all the tolerant wild species exhibited high tissue tolerance score than that of the cultivated tolerant checks as well as the sensitive check IR29. The wild species, *O*. *rhizomatis* had the highest tissue tolerance score (LC_50_ = 4.5 mg/g) among all wild-tolerant lines and is followed by *O*. *eichingeri* (LC_50_ = 3.4 mg/g) (**Figure 6B**).

### Na^+^ Content from Leaf Washing Solution

The sodium ions concentration released through leaf washing was calculated per mm^2^ of the leaf from the wild-tolerant species and the check lines. The Na^+^ content was very low in leaf-washed solution from all wild-tolerant materials, except for *O. coarctata* (Additional File 4: Supplementary Figure S3). The Na^+^ content released from *O*. *coarctata* was 0.50% which was dramatically higher (*p* < 0.001) than the remaining genotypes and suggested Na^+^ ion extrusion from leaf surface. To observe the leaf surface of the wild-tolerant species, leaf anatomy was conducted. The salt exuding gland was observed only in the leaves of *O*. *coarctata* (Additional File 5: Supplementary Figure S4).

## Discussion

Currently limited salt-tolerant genotypes are available in the cultivated species of *O*. *sativa* and *O*. *glabberrima*, and those genotypes have been extensively used in rice breeding. The salt-tolerant cultivar, Pokkali is one such example which has been used as a potential donor in several rice breeding programs (Waziri et al., 2016). The narrow genetic diversity of the cultivated rice with a handful source of salt-tolerant donors is a major limitation for further augmentation of salt tolerance trait in the elite rice varieties. Apart from the two cultivated rice species, *O. sativa* and *O. glaberrima*, the rice germplasm has 22 different wild species. Compared to the cultivated rice, the wild species of rice has a wide genetic diversity. Today’s cultivated rice is the result of a long-term domestication process of the wild ancestor and during which many of the valuable genes are thought to have been lost. It is estimated that only 10–20% of the wild species diversity is present in cultivated rice (Zhu et al., 2007; Palmgren et al., 2014). To develop a climate resilient agriculture for sustainable rice production, time has come to go back to the wild progenitors to capture the untapped traits and improve rice breeding with exotic genes. While the wild rice germplasm has been identified with carrying different resistance/tolerance factor for various stress, a very limited knowledge has been acquired for salt tolerance. To identify salt-tolerant lines in the wild AA genome, Akbar et al. (1987) carried out salinity screening in seven wild rice species along with two cultivated species in a hydroponic experiment system but none of the wild species accessions tested was found to be as tolerant as the cultivated landrace Nona Bokra. The salt tolerance of *O*. *coarctata* (KKLL genome) is well documented from several previous studies (Bal and Dutt, 1986; Jena, 1994; Sengupta and Majumder, 2010; Menguer et al., 2017). Salt tolerance in other wild species like *O. punctata, O. officinalis*, and *O. rufipogon* was also reported (Farooq et al., 1992; Tian et al., 2011; Mishra et al., 2016; Zhou et al., 2016). In this study, we simultaneously tested all 22 wild species together with the cultivated checks at a high NaCl concentration (240 mM NaCl) compared to the earlier screening conditions to identify salt-tolerant species and to understand their salt tolerance mechanism through physiological, biochemical, and molecular studies. Except for *O*. *coarctata*, six wild rice species were newly identified in this study (**Table 1**). But the previously isolated tolerant species did not show salt tolerance in this study probably due to use of different accessions of the species or a different screening conditions. Around 4,370 accessions of wild species are available in the IRRI gene bank. Hence, future research can include more accessions for salinity screening and it can get more salt-tolerant sources for further improvement of rice cultivars.

One of the interesting result came out from the analysis of ionic content was the occurrence of high Na^+^ and high Na^+^/K^+^ ratio in the young leaves of novel salt-tolerant germplasm, an opposite ionic paradigm in tolerant cultivars. Salt tolerance trait in rice cultivars is likely to be dependent on their ability to maintain low Na^+^ concentration in young leaves through Na^+^ exclusion (Lin et al., 2004; Thomson et al., 2010; Platten et al., 2013). Consistent to earlier research, this study also found low Na^+^ in shoot and high in root in the cultivated salt-tolerant lines implying that Na^+^ has been actively excluded through the root. In contrast, the novel salt-tolerant wild species accessions accumulated high Na^+^ and showed high Na^+^/K^+^ ratio in shoot. For the wild species-tolerant lines, the high Na^+^ and Na^+^/K^+^ ratio per unit dry mass in the shoot than root implies a poor Na^+^ exclusion process unlike tolerant cultivars. Wild accessions of *O. eichingeri* and *O. minuta* had low shoot/root Na^+^ but it was not low as cultivated salt-tolerant lines. The high ionic content in wild-tolerant species suggested us to check for the presence of the three key Na^+^ excluding genes *OsSOS1, OsHKT1;5, OsHKT1;4*, and one Na^+^ sequestration gene *OsNHX1* in wild-tolerant species and to observe their expression pattern under salt stress. From PCR amplifications with genomic DNA, it was found that the primers binding to the intronic regions did not generate the PCR products (e.g., OsHF1, OsHF2, OsHF3, and OsHF4 primer sets for *OsHKT1;5* and SOS1F2 for *OsSOS1*). In contrast, most of the primers binding to the exonic regions produced expected PCR amplicon in the wild species like in the checks, except for the OsH4F1 primer set for *OsHKT1;4* (Additional File 3: Supplementary Figure S2B). These PCR results indicate that the nucleotide variations are very high in intronic regions compared to exonic regions between the cultivated and wild rice species. Characterization of the gene sequences and comparisons between the sensitive and tolerant accessions will be required to understand the functional nucleotides for salt tolerance in the future.

For further gene expression analysis, all three CCDD genome species with the checks were used. Unexpectedly, *OsHKT1;5* and *OsSOS1* were strongly transcribed by salt stress in both cultivated and wild salt-tolerant accessions. And also similar expression pattern of these two genes were observed in the salt-sensitive cultivar IR29. However, Na^+^ accumulation pattern was different among the samples tested, although all samples showed same expression patterns of the Na^+^ excluding genes in root under salt stress condition. Platten et al. (2013) reported 10 allele types of *OsHKT1;*5 from the diverse cultivated accessions through PCR-Sanger sequencing method and showed the different level of Na^+^ exclusion among the different allele types in root. Similarly, the sequence difference in coding regions of the *OsHKT1;5* and *OsSOS1* genes among accessions in this study might cause different protein sequence, resulting in differential Na^+^ accumulation patterns. For *OsHKT1;4*, we detected a high expression in the leaf of all CCDD wild species at the seedling growth stage both in normal and stress conditions while *OsHKT1;4* expression was very low in the cultivated checks. Moreover, *OsHKT1;4* expression level increased for *O. latifolia* (**Figure 5B**) under salt stress. One of the distinctive features of *OsHKT1;4* is its steady-state expression in the leaf sheaths in Nipponbare throughout the whole growth stage. In salt-stressed Nipponbare, *OsHKT1;4*-mediated transport contributes for Na^+^ homeostasis for the reproductive growth phase rather than vegetative growth (Suzuki et al., 2016). We assume that the *OsHKT1;4* expression in the leaf tissue of wild species may contribute to Na^+^ exclusion from the leaf and its further sequestration in the vacuole or to the apoplast space. The *OsNHX1* gene is known to play a key role in the vacuolar compartmentalization (Yamaguchi et al., 2013). It has been shown in many plant species that over expression of *OsNHX1* gene confers salt tolerance (Apse et al., 1999; Zhang et al., 2001; Agarwal et al., 2013). In the study, *OsNHX1* expression was increased at the root under salt stress in case of cultivated checks implying Na^+^ sequestration in the root vacuole. This may be one of the reasons for getting a high Na^+^ content in the roots of tolerant cultivated checks. *OsNHX1* did not express in any of the CCDD genome species both at normal and stress (**Figure 5A**). This result suggests that excess Na^+^ in these species might be sequestrated in vacuole without assistance of *OsNHX1* gene. It has been also reported that the Na^+^ uptake to vacuole can also be possible through pinocytosis, which is an energy efficient way without requiring Na^+^/H^+^ exchange activity in halophytes (Shabala and Mackay, 2011). In this process, the invagination of tonoplast can engulf the apoplastic Na^+^ and move it to vacuole. Further study is required to elucidate the sequestration mechanism in leaf for tissue tolerance.

Tissue tolerance is also an important mechanism for salinity tolerance in crop plant. Tissue tolerance is defined as the ability of cell or tissues to tolerate internal Na^+^ and Cl^-^. A number of factor can contribute to maintain tissue tolerance such as compartmentalization of excess Na^+^ in the vacuole keeping its cytosolic concentration at much low level, adequate translocation of K^+^, adjustment of osmotic pressure, and regulation of ROS (Munns et al., 2016). Osmotic adjustment is essential for plant to maintain the turgor pressure and prevent water loss during salinity stress. Since at the elevated Na^+^ and Na^+^/K^+^ ratio, the newly identified tolerant wild species showed a better morphology and we hypothesize that these high Na^+^ would have benefited in the adjustment of osmotic pressure. A large portion of the osmotic adjustment is carried by the synthesis of organic solutes which is an energy requiring process (Munns and Gilliham, 2015). Alternatively, the high accumulation of Na^+^ in the cellular organelle like vacuole may function like osmoticum to maintain osmotic balance (Maathuis et al., 2014; Shabala and Pottosin, 2014). This energy-efficient strategies are used by halophytes (Flowers and Colmer, 2008) and salt-tolerant non-halophyte plant like barley. The indication of the probable involvement of tissue tolerance also came from MDA assay. The level of lipid peroxidation has been widely used as an indicator of ROS-mediated damage to cell membranes under stressful conditions (Borsani et al., 2001; Apel and Hirt, 2004). Increase in lipid peroxidation under salinity stress associates with increased production of ROS. MDA is one of the final products of peroxidation of unsaturated fatty acids in phospholipids and is responsible for cell membrane damage. Plants with low accumulation of MDA under stress condition are more adaptable to the stress than high MDA. Especially, the wild-tolerant species had the lowest level of MDA, suggesting the proper regulation of ROS through various scavenging mechanisms. Another indication of the tissue tolerance came from the observation that wild salt-tolerant species accumulated more Na^+^ in their leaves with the less chlorophyll loss. Loss of chlorophyll pigment can lead to photosynthesis decline. Retention of high chlorophyll in the leaves under salinity is an indication of tissue tolerance (Yeo and Flowers, 1986; Munns et al., 2016). In our study, we exclusively estimated the chlorophyll level in the young emerging leaves (L6) under the salt stress and its reduction from the control condition. In the cultivated tolerant checks, the chlorophyll reduction was very high even higher than the sensitive check IR29. However, chlorophyll level in wild-tolerant lines undergoes less reduction under salinity. Salinity toxic symptoms such as chlorosis, leaf rolling, and meristem dehydration were more apparent in salinity-tolerant cultivars than the wild relatives. The third evidence for tissue tolerance came from Na^+^ tissue tolerance assay. All the wild species were found to have a high Na^+^ tissue tolerance which means a 50% of chlorophyll degradation occurs at a higher concentration of Na^+^ which is an opposite phenomenon in cultivated tolerant lines. In the case of cultivated tolerant lines, the 50% reduction of chlorophyll occurred in spite of having a low Na^+^ concentration in their leaves. The increased Na^+^ ions in shoot without apparent loss of photosynthetic function and sustained cellular integrity resulting to unhampered growth suggest tissue tolerance as the primary means under saline perturbation.

To observe other possible component of salt tolerance we investigated the role of plant vigor and salt excretion from leave surface. Vigorous growth with larger biomass provides salt dilution and maintains low tissue Na^+^ concentration that helps the plant to survive under salt stress (Yeo et al., 1990). Breeding more vigorous plant for saline-affected soil was suggested to get more yields (Richards, 1992). In rice, most of the traditional salt-tolerant landraces are vigorous in growth (Yeo et al., 1990). Similarly, in our study a strong positive correlation was observed between vigor score and salt tolerance in the cultivated tolerant lines. However, on the contrary vigor score was not correlated with salt tolerance in the tolerant wild species. Na^+^ exudation from the leaf through pore-like structures such as hydathode and salt glands lowers the cytosolic Na^+^ and prevents chlorophyll loss (Bal and Dutt, 1986; Flowers et al., 1990; Negrão et al., 2011). To know whether any other wild species use this component of salinity tolerance mechanism, we measured ion content from leaf-washed solution from all genotypes. None of the genotypes, except for *O*. *coarcctata*, accumulate Na^+^ outside the leaves and salt glands were not observed in their leaves suggesting this mechanism of salt tolerance is absent in other tolerant wild accessions.

The phloem recirculation may also be a mechanism of salt tolerance where Na^+^ in the leaf can be redistributed to root via phloem. This phloem recirculation is not a well-established mechanism in rice although *OsHK2;1* located in shoot vascular bundle is believed to have a role. The result from using positron-emitting tracer imaging system which can trace the direction of Na^+^ transport, it was found that the Na^+^ accumulates in shoot only (Fujimaki et al., 2015). In our study, a low Na^+^ content was detected in wild-tolerant species roots which suggest no noticeable role of Na^+^ recirculation via phloem.

Our study revealed availability of new salt tolerance sources in the wild germplasm which will be certainly helpful for the future rice breeding and biotechnology research. In addition to the mechanism study of salt tolerance using the newly isolated wild rice species, utilization of the trait is also crucial to improve salt tolerance of the cultivated rice varieties. For this, development of introgression lines (ILs) having the wild rice chromosome segments, screening of the ILs against salt stress, and identifications of QTL/gene using the ILs are required in the future. The QTLs/gene(s) can be easily transferred to the rice varieties through the marker-assisted breeding (MAB). And also the additive effects between the conventional salt tolerance genes and the newly identified genes derived from wild species need to be tested. Alternatively, the advanced genomics tools can be employed for rapid validation of the genes. Sequence comparisons of the major salt tolerance genes between salt sensitive and tolerant species are one of priorities to find a commonality like functional nucleotide polymorphisms (FNPs) causing the trait. Finally, this can be confirmed by direct-transferring of the target genes from wild rice species to rice varieties using transgenic method. This work will be valuable to isolate superior alleles of the known genes.

## Author Contributions

KJ, MP, MD, and S-RK conceived the idea and designed the research. MP, RV, JE, and FE conducted the experiment. KJ, MP, and S-RK wrote the manuscript. All authors contributed to the conceptualization of the study, read, and approved the final version of the manuscript.

## Conflict of Interest Statement

The authors declare that the research was conducted in the absence of any commercial or financial relationships that could be construed as a potential conflict of interest.

## Abbreviations

DMRT: Duncan’s multiple range test
DSS: days of seedling survival
IRRI: International Rice Research Institute
MDA: malondialdehyde
QTL: quantitative trait loci
ROS: reactive oxygen species
SES: standard evaluation system
VI: vigor score
VSI: visual salt injury
XPC: xylem perenchyma cells
