# Supplementary material for: Newly Identified Wild Rice Accessions Conferring High Salt Tolerance Might Use a Tissue Tolerance Mechanism in Leaf
Source: Front Plant Sci. 2018 Apr 23;9:417. doi: 10.3389/fpls.2018.00417 (PMC5926390; doi:10.3389/fpls.2018.00417)
Supplement: Supplementary file 1 [file Presentation_1.pptx]

## Slide 1
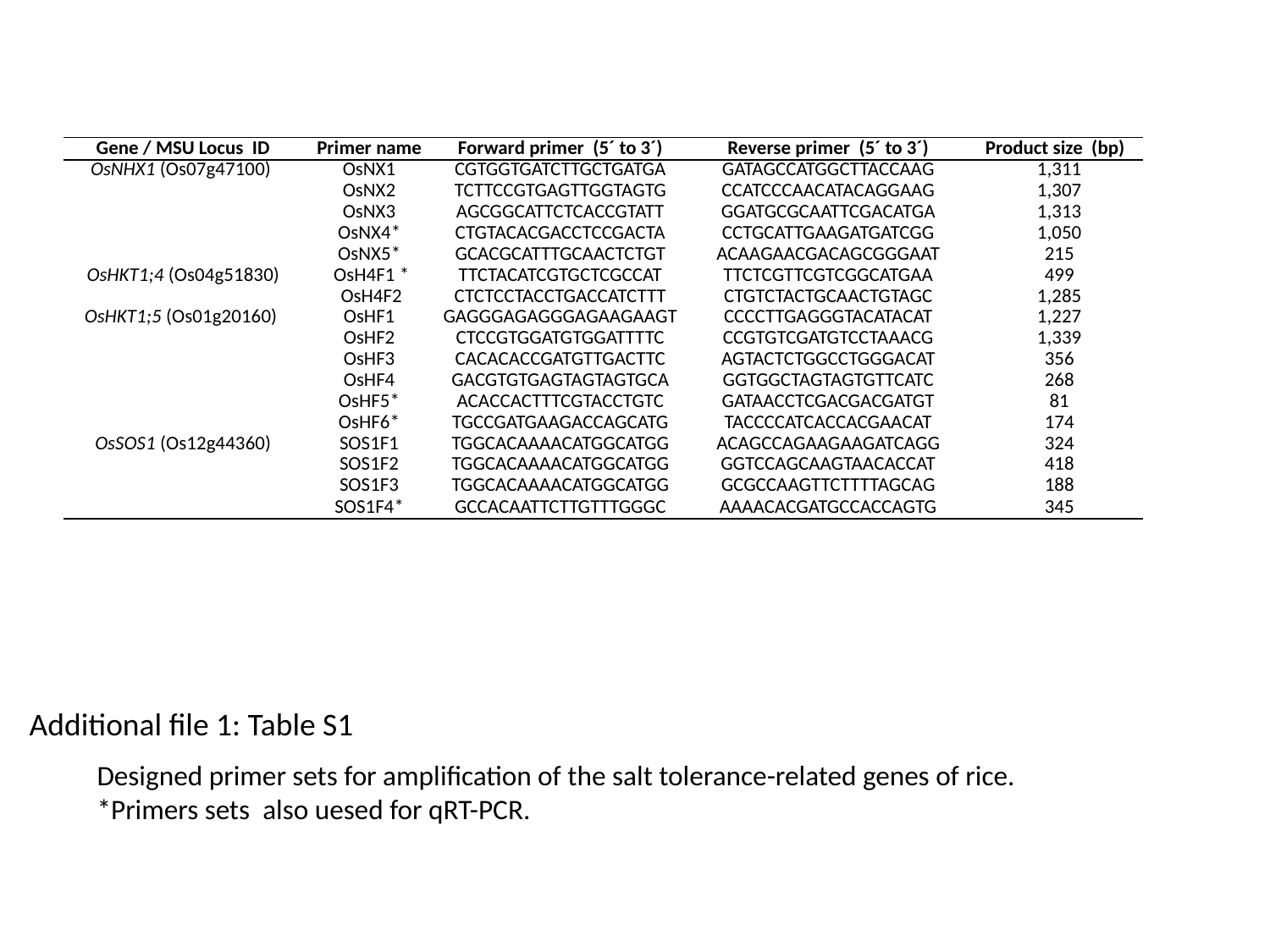

| Gene / MSU Locus ID | Primer name | Forward primer (5´ to 3´) | Reverse primer (5´ to 3´) | Product size (bp) |
| --- | --- | --- | --- | --- |
| OsNHX1 (Os07g47100) | OsNX1 | CGTGGTGATCTTGCTGATGA | GATAGCCATGGCTTACCAAG | 1,311 |
| | OsNX2 | TCTTCCGTGAGTTGGTAGTG | CCATCCCAACATACAGGAAG | 1,307 |
| | OsNX3 | AGCGGCATTCTCACCGTATT | GGATGCGCAATTCGACATGA | 1,313 |
| | OsNX4\* | CTGTACACGACCTCCGACTA | CCTGCATTGAAGATGATCGG | 1,050 |
| | OsNX5\* | GCACGCATTTGCAACTCTGT | ACAAGAACGACAGCGGGAAT | 215 |
| OsHKT1;4 (Os04g51830) | OsH4F1 \* | TTCTACATCGTGCTCGCCAT | TTCTCGTTCGTCGGCATGAA | 499 |
| | OsH4F2 | CTCTCCTACCTGACCATCTTT | CTGTCTACTGCAACTGTAGC | 1,285 |
| OsHKT1;5 (Os01g20160) | OsHF1 | GAGGGAGAGGGAGAAGAAGT | CCCCTTGAGGGTACATACAT | 1,227 |
| | OsHF2 | CTCCGTGGATGTGGATTTTC | CCGTGTCGATGTCCTAAACG | 1,339 |
| | OsHF3 | CACACACCGATGTTGACTTC | AGTACTCTGGCCTGGGACAT | 356 |
| | OsHF4 | GACGTGTGAGTAGTAGTGCA | GGTGGCTAGTAGTGTTCATC | 268 |
| | OsHF5\* | ACACCACTTTCGTACCTGTC | GATAACCTCGACGACGATGT | 81 |
| | OsHF6\* | TGCCGATGAAGACCAGCATG | TACCCCATCACCACGAACAT | 174 |
| OsSOS1 (Os12g44360) | SOS1F1 | TGGCACAAAACATGGCATGG | ACAGCCAGAAGAAGATCAGG | 324 |
| | SOS1F2 | TGGCACAAAACATGGCATGG | GGTCCAGCAAGTAACACCAT | 418 |
| | SOS1F3 | TGGCACAAAACATGGCATGG | GCGCCAAGTTCTTTTAGCAG | 188 |
| | SOS1F4\* | GCCACAATTCTTGTTTGGGC | AAAACACGATGCCACCAGTG | 345 |
Additional file 1: Table S1
Designed primer sets for amplification of the salt tolerance-related genes of rice.
*Primers sets also uesed for qRT-PCR.

## Slide 2
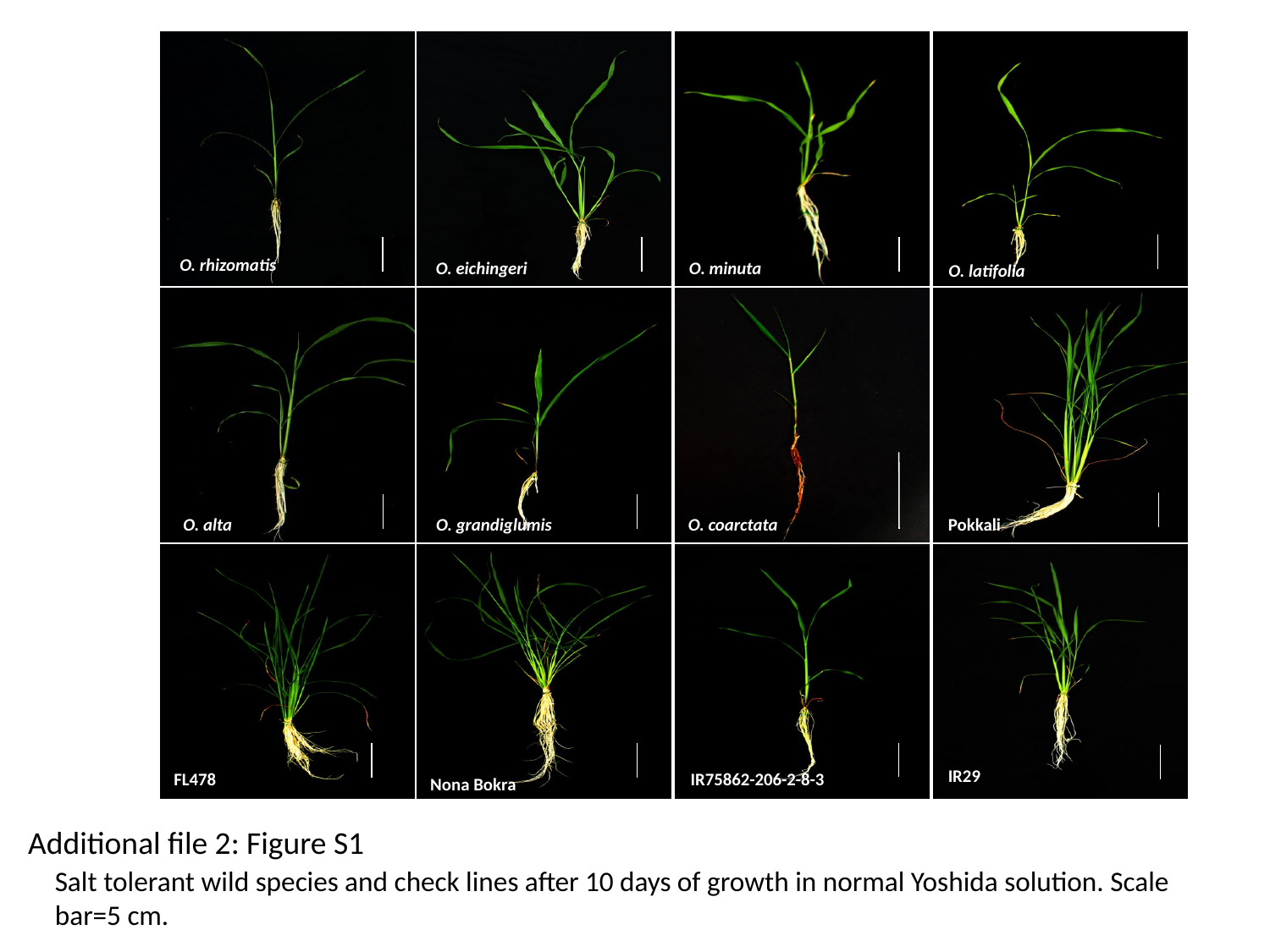

O. rhizomatis
O. eichingeri
O. minuta
O. latifolia
O. alta
O. grandiglumis
O. coarctata
Pokkali
IR29
IR75862-206-2-8-3
FL478
Nona Bokra
Additional file 2: Figure S1
Salt tolerant wild species and check lines after 10 days of growth in normal Yoshida solution. Scale bar=5 cm.

## Slide 3
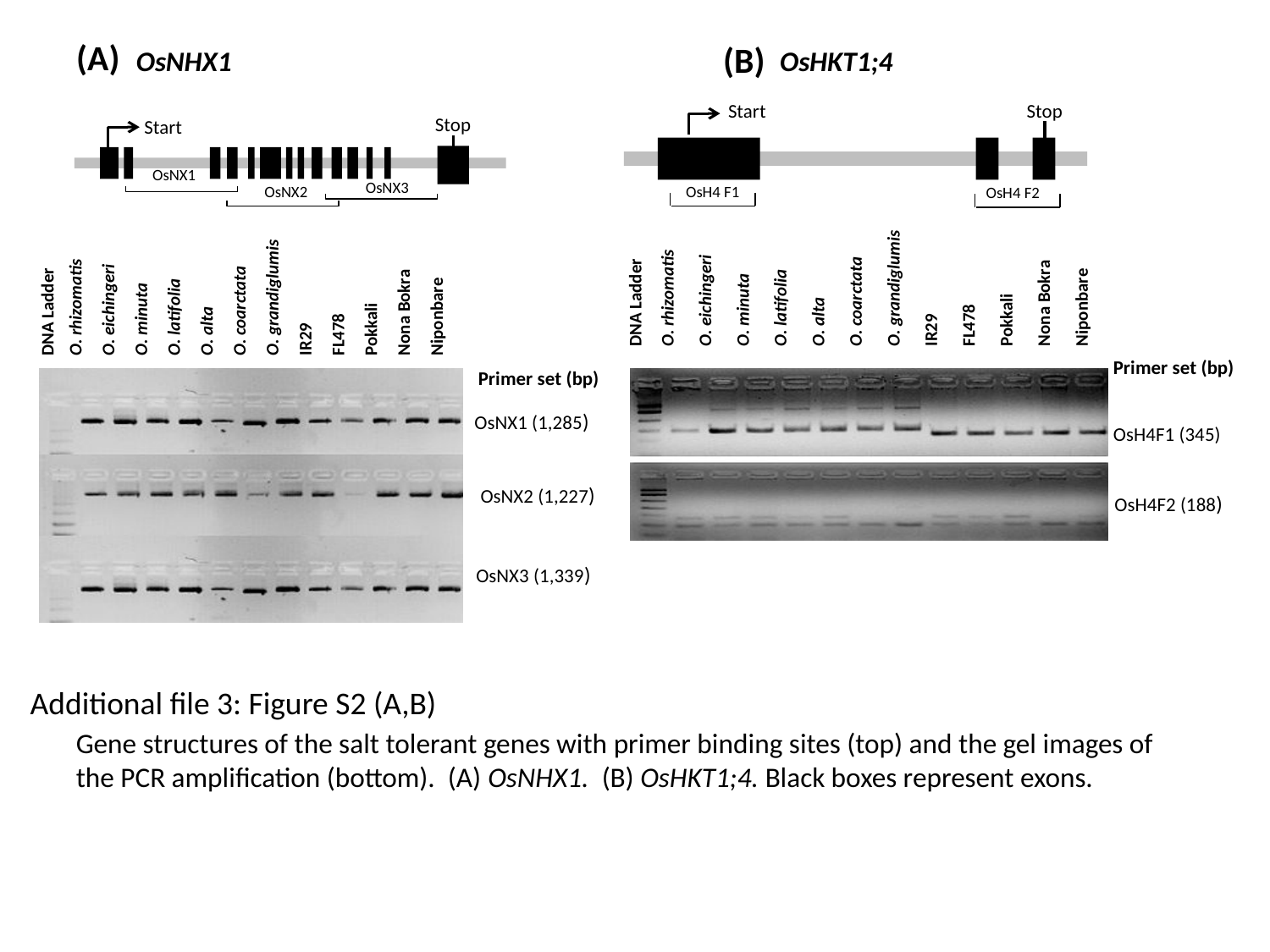

(A)
(B)
OsHKT1;4
OsNHX1
Start
Stop
Stop
Start
OsNX1
OsNX3
OsH4 F1
OsNX2
OsH4 F2
| DNA Ladder | O. rhizomatis | O. eichingeri | O. minuta | O. latifolia | O. alta | O. coarctata | O. grandiglumis | IR29 | FL478 | Pokkali | Nona Bokra | Niponbare |
| --- | --- | --- | --- | --- | --- | --- | --- | --- | --- | --- | --- | --- |
| DNA Ladder | O. rhizomatis | O. eichingeri | O. minuta | O. latifolia | O. alta | O. coarctata | O. grandiglumis | IR29 | FL478 | Pokkali | Nona Bokra | Niponbare |
| --- | --- | --- | --- | --- | --- | --- | --- | --- | --- | --- | --- | --- |
Primer set (bp)
Primer set (bp)
OsNX1 (1,285)
OsNX2 (1,227)
OsNX3 (1,339)
OsH4F1 (345)
OsH4F2 (188)
Additional file 3: Figure S2 (A,B)
Gene structures of the salt tolerant genes with primer binding sites (top) and the gel images of the PCR amplification (bottom). (A) OsNHX1. (B) OsHKT1;4. Black boxes represent exons.

## Slide 4
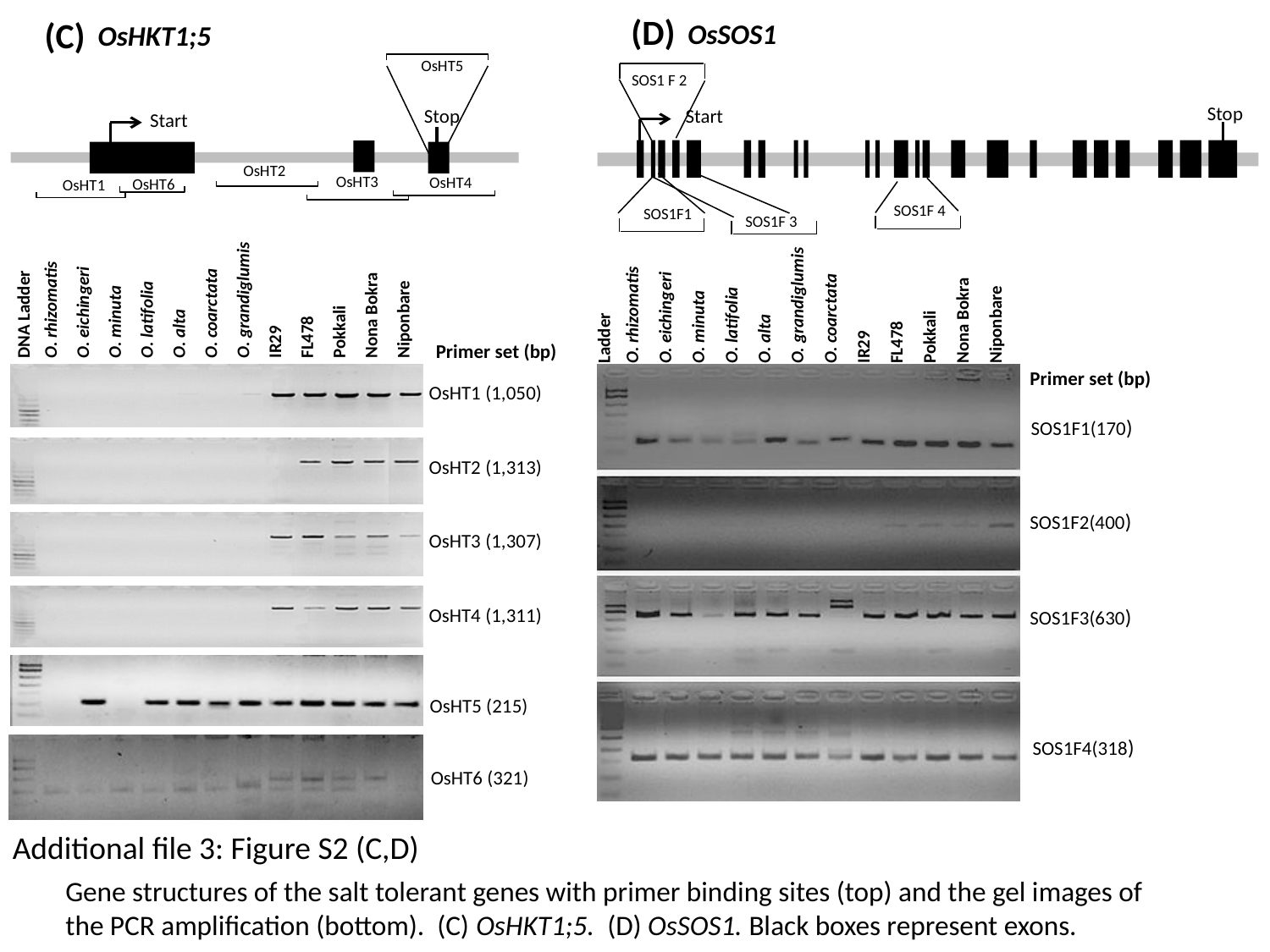

(D)
OsSOS1
(C)
OsHKT1;5
OsHT5
SOS1 F 2
Stop
Start
SOS1F 4
SOS1F1
SOS1F 3
Stop
Start
OsHT2
OsHT3
OsHT4
OsHT6
OsHT1
| DNA Ladder | O. rhizomatis | O. eichingeri | O. minuta | O. latifolia | O. alta | O. coarctata | O. grandiglumis | IR29 | FL478 | Pokkali | Nona Bokra | Niponbare |
| --- | --- | --- | --- | --- | --- | --- | --- | --- | --- | --- | --- | --- |
| Ladder | O. rhizomatis | O. eichingeri | O. minuta | O. latifolia | O. alta | O. grandiglumis | O. coarctata | IR29 | FL478 | Pokkali | Nona Bokra | Niponbare |
| --- | --- | --- | --- | --- | --- | --- | --- | --- | --- | --- | --- | --- |
Primer set (bp)
Primer set (bp)
OsHT1 (1,050)
SOS1F1(170)
OsHT2 (1,313)
SOS1F2(400)
OsHT3 (1,307)
OsHT4 (1,311)
SOS1F3(630)
OsHT5 (215)
SOS1F4(318)
OsHT6 (321)
Additional file 3: Figure S2 (C,D)
Gene structures of the salt tolerant genes with primer binding sites (top) and the gel images of the PCR amplification (bottom). (C) OsHKT1;5. (D) OsSOS1. Black boxes represent exons.

## Slide 5
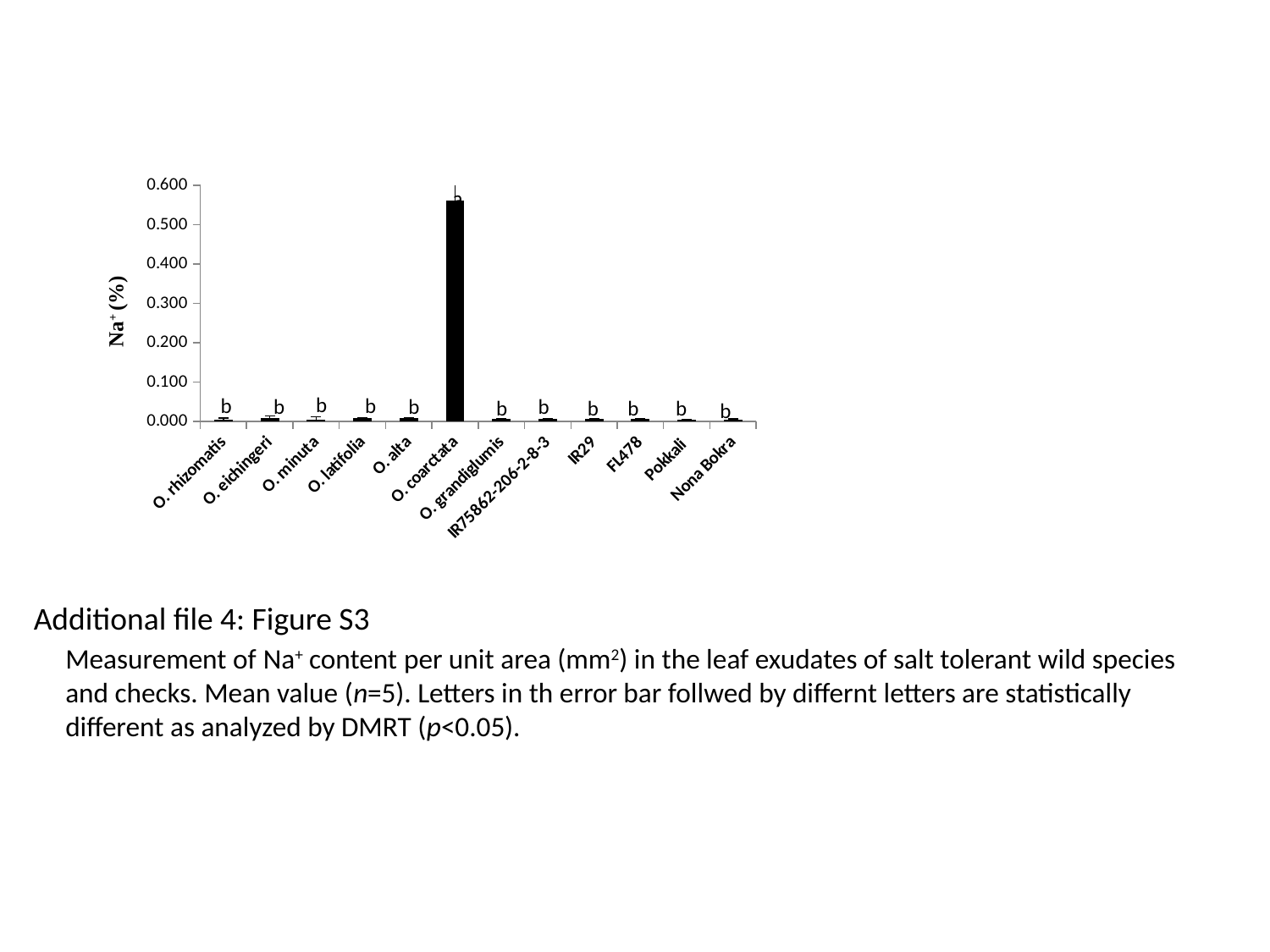

### Chart
| Category | |
|---|---|
| O. rhizomatis | 0.005000000000000004 |
| O. eichingeri | 0.008000000000000009 |
| O. minuta | 0.005000000000000004 |
| O. latifolia | 0.00900000000000001 |
| O. alta | 0.008000000000000009 |
| O. coarctata | 0.5624956789333336 |
| O. grandiglumis | 0.006000000000000006 |
| IR75862-206-2-8-3 | 0.006000000000000006 |
| IR29 | 0.005375787104207128 |
| FL478 | 0.0058571428571428585 |
| Pokkali | 0.004914727117052639 |
| Nona Bokra | 0.005114285714285714 |a
Na+ (%)
b
b
b
b
b
b
b
b
b
b
b
Additional file 4: Figure S3
Measurement of Na+ content per unit area (mm2) in the leaf exudates of salt tolerant wild species and checks. Mean value (n=5). Letters in th error bar follwed by differnt letters are statistically different as analyzed by DMRT (p<0.05).

## Slide 6
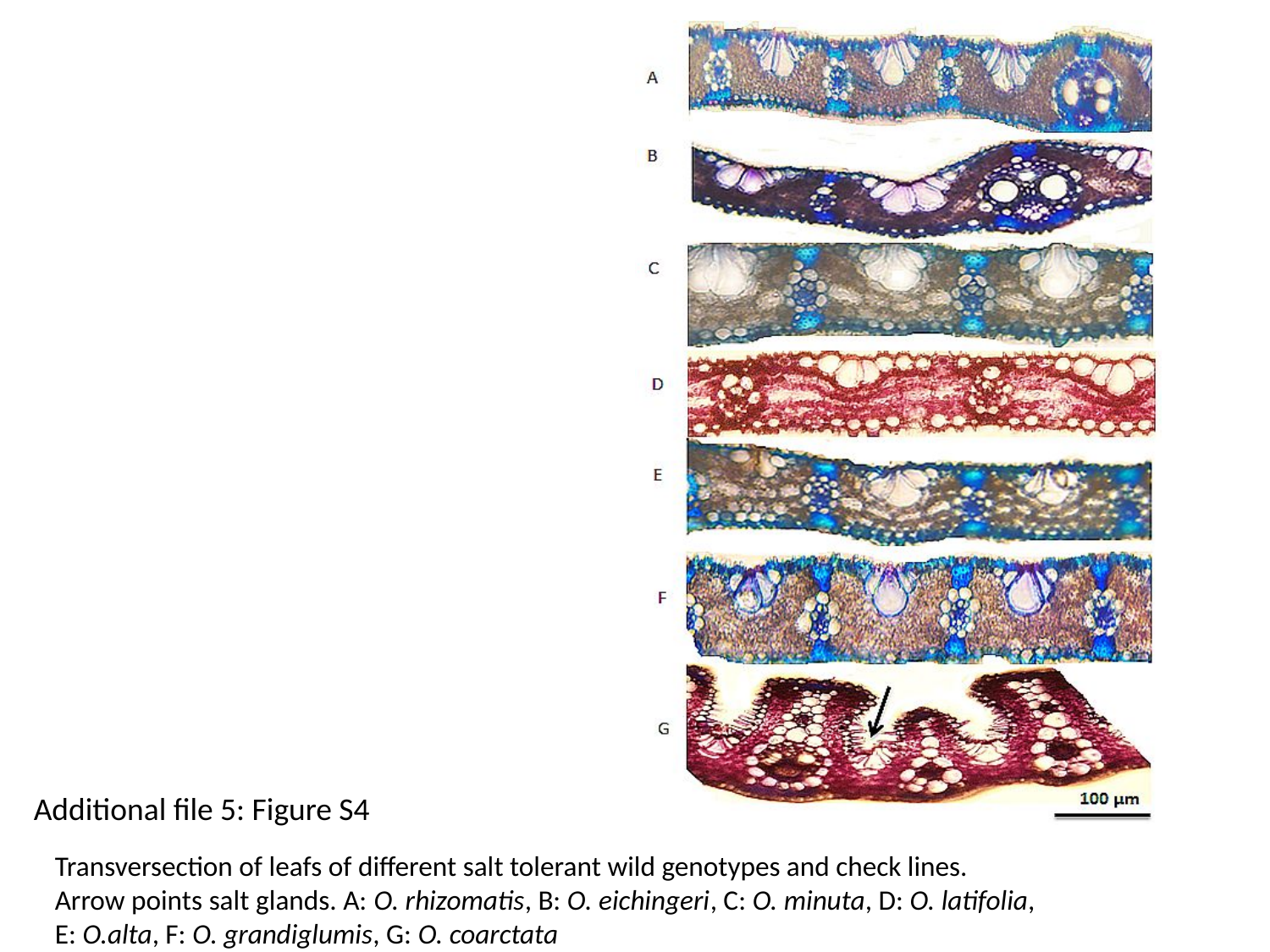

Additional file 5: Figure S4
Transversection of leafs of different salt tolerant wild genotypes and check lines. Arrow points salt glands. A: O. rhizomatis, B: O. eichingeri, C: O. minuta, D: O. latifolia, E: O.alta, F: O. grandiglumis, G: O. coarctata
